# Platelet Releasate Injection as a Novel Treatment for Ulnar Neuritis at the Elbow: A Case Report

**DOI:** 10.7759/cureus.42223

**Published:** 2023-07-20

**Authors:** Michael C Bejarano, Daniel A Clearfield

**Affiliations:** 1 Medicine, Texas College of Osteopathic Medicine at University of North Texas Health Science Center, Fort Worth, USA; 2 Sports Medicine, Motion Is Medicine Sports Medicine, North Richland Hills, USA

**Keywords:** ultrasound-guided perineural injection, ultrasound-guided intraneural injection, nerve repair techniques, physical medicine and rehabilitation, sports medicine, hydrodissection, platelet-rich plasma, platelet releasate, ulnar nerve entrapment, ulnar neuritis

## Abstract

This report examines the efficacy of platelet releasate injection as a treatment method for ulnar neuritis. Platelet-rich plasma (PRP), an autologous product of concentrated platelets, has the potential to accelerate healing in injured peripheral nerves by releasing growth factors that promote nerve repair. Platelet releasate, the supernatant of thrombin-activated PRP, has yet to be thoroughly investigated as a treatment option for ulnar neuritis.

In this report, a 42-year-old female patient presented with right-sided elbow and neck pain that was subsequently diagnosed as ulnar neuritis and neurogenic thoracic outlet syndrome. Initial imaging at the right elbow demonstrated ulnar nerve entrapment within the arcade of Struthers. The patient’s symptoms were first managed with home exercise and ulnar nerve hydrodissection at the elbow, which decreased but did not resolve her pain. Platelet releasate injection of the ulnar nerve at the elbow was subsequently performed. Six weeks post-procedure, the patient reported additional pain improvement. Provocative tests at the elbow were negative and imaging demonstrated a normal-appearing ulnar nerve. Despite these results, the patient was not completely symptom-free; persistent symptoms were attributed to her concomitant neurogenic thoracic outlet syndrome.

While platelet releasate injection has not previously been explored as a treatment option for ulnar neuritis, this case demonstrates how platelet releasate injection may facilitate healing in an ulnar nerve injured by entrapment. Further investigation could support platelet releasate injection as an effective monotherapy or as an adjunct treatment for ulnar neuritis and similar peripheral neuropathies.

## Introduction

Ulnar neuritis is a peripheral neuropathy in the upper extremity that results from inflammation of the ulnar nerve secondary to causes such as injury, infection, or compression of the nerve. Ulnar nerve entrapment, a compressive neuropathy that can lead to ulnar neuritis, is the second-most common entrapment neuropathy next to median nerve entrapment at the carpal tunnel [1]. Ulnar nerve compression commonly occurs in patients who frequently maintain prolonged elbow flexion or prolonged wrist dorsiflexion, often due to repetitive occupational tasks [1]. Repetitive work such as typing on a keyboard for extended periods of time, which involves flexing the elbow while compressing it against a surface, particularly elevates the risk of ulnar nerve compression due to increased intraneural pressure [1]. Patients with ulnar nerve compression usually present with paresthesia of the fourth and fifth digits and may present with pain localized to the medial aspect of the elbow at the epicondylar groove [1]. Chronic cases from prolonged compression can lead to intrinsic muscle weakness: there may be noticeable wasting of the muscles of the hands and forearm [1]. Motor symptoms include weak grip, decreased pinch strength, and difficulty with fine motor tasks [1].

Electromyography and nerve conduction studies are the standard of care for identifying evidence of peripheral nerve compression and establishing the diagnosis of profound ulnar nerve injury [2]. Diagnosis of ulnar neuritis may also be determined by radiographs if bony abnormalities are suspected as the cause or by magnetic resonance imaging if a soft tissue anomaly is the culprit [1]. Ultrasound of the ulnar nerve is beneficial for observing potential nerve injury, as the nerve may be visualized as enlarged, crushed, flattened, and/or damaged with reduced echogenicity [1].

Conservative therapy for ulnar neuritis at the elbow includes modifying activity, putting protective soft padding over the medial aspect of the elbow, and/or placing the elbow in a splint to decrease elbow flexion [3]. If conservative management fails, the definitive treatment is surgical intervention, which carries the risk of damage to surrounding nerves and blood vessels. Possible complications of surgery at the elbow to resolve ulnar nerve entrapment include hematoma, incomplete release of all potential points of compression, injury to the medial antebrachial cutaneous nerve, and injury to the ulnar nerve or the flexor carpi ulnaris branches [4].

Another possible treatment for neuropathic pain relief is peripheral nerve stimulation. A peripheral nerve stimulator is composed of a single electrode with several contact leads; the electrode is implanted near the target peripheral nerve, and a battery is used to stimulate the electrode [5]. Peripheral nerve stimulation carries the risk of both hardware-related and biological complications. Potential hardware complications involve lead-related issues such as lead migration, fracture, or malfunction, as well as problems related to the implantable pulse generator (IPG), including battery failure or recharging difficulties [6]. Potential biological complications include infection, skin erosion, and nerve damage [6].

A minimally invasive technique that has emerged as a treatment option for peripheral nerve entrapment is hydrodissection. Hydrodissection is a technique that involves injecting a solution, most commonly dextrose 5% in water (D5W), perineurally around an entrapped nerve. This process helps to separate the nerve from the surrounding tissue and adjacent structures that may be compressing it. The therapeutic benefit of hydrodissection lies in its capacity to relieve compression and allow the entrapped nerve to return to its original form and function. In this case, D5W hydrodissection was used in the patient’s treatment to separate the ulnar nerve from the medial epicondyle, arcade of Struthers, and surrounding tissue.

Limited research has been conducted on the restorative effects of platelet releasate, the supernatant of platelet-rich plasma (PRP), as a treatment modality for injured peripheral nerves. While current applications of PRP include therapies that enhance healing for tendinopathies and muscle or ligament tears, PRP has demonstrated potential in promoting nerve repair [7]. Ultrasound-guided PRP injections for peripheral nerve crush injuries have demonstrated accelerated recovery of axonal function and dampened atrophy of the target muscle, indicating that ultrasound-guided PRP injection may be a widely applicable treatment modality for peripheral nerve injury [8]. Investigations on the effects of PRP on injured nerves have illustrated that PRP may enhance nerve regeneration through sustained delivery of growth factors [8]. These growth factors impact several processes of nerve repair, including angiogenesis, chemotaxis, cell proliferation, and extracellular matrix accumulation [8].

Thus, the therapeutic potential of PRP is associated with the release of biomolecules and growth factors stored in platelets that can facilitate nerve regeneration [7]. By utilizing a platelet-activating factor such as thrombin to promote the degranulation of platelets within PRP, the subsequent platelet releasate that is formed contains a greater concentration of growth factors that may enhance nerve repair. Injecting platelet releasate within an injured nerve and its surrounding environment provides targeted delivery of a pool of biomolecules and growth factors that has the potential to augment healing of the nerve tissue.

The clinical application of platelet releasate for healing injured peripheral nerves has yet to be firmly established: current data are sparse on the efficacy of platelet releasate injection for the treatment of compression neuropathies. To our knowledge, platelet releasate injection has yet to be examined as a potential treatment option for ulnar neuritis. The aim of this case report was to investigate the previously unexplored approach of using platelet releasate injection to treat ulnar neuritis. Additionally, it aims to provide preliminary evidence for the potential efficacy of this method. This report considers the novel approach of using intraneural and perineural platelet releasate injection in conjunction with D5W hydrodissection for treating ulnar neuritis due to ulnar nerve entrapment at the elbow.

## Case presentation

A 42-year-old ambidextrous female presented to a sports medicine clinic with right-sided neck, elbow, and forearm pain. The pain had been persistent for a duration of three months. The patient endorsed paresthesia localized at the elbow and radiating to the forearm and fourth and fifth digits. Slight weakness was noted in right-hand grip strength. The patient’s pain was worsened by repetitive computer work and overuse from lifting heavy objects in her occupation as a labor and delivery nurse. She had been consistently wearing an arm brace and applying diclofenac gel to the affected areas for five weeks, which provided some mild relief but did not resolve symptoms. The patient had previously seen an orthopedic surgeon, who diagnosed the patient’s condition as ulnar nerve entrapment and offered surgical intervention. The patient declined surgery and sought alternative minimally invasive treatments.

Upon presentation to the sports medicine clinic, the diagnosis of ulnar nerve entrapment as well as right-sided neurogenic thoracic outlet syndrome (nTOS) was given at the sports medicine clinic through history and physical, and further characterized through diagnostic neurosonography. The patient experienced pain on palpation of the right elbow proximal to the cubital tunnel within the arcade of Struthers at the fascial dip. Valgus and varus stress tests of the right elbow, which evaluate for possible injury to the medial collateral ligament and the lateral collateral ligament, respectively, were both negative. Ulnar nerve compression test and Tinel’s test at the elbow, which evaluate for ulnar nerve irritation, were both positive. Froment’s sign, which evaluates for ulnar nerve palsy, was also positive. These positive tests on physical examination further established the diagnosis.

**Figure 1:**
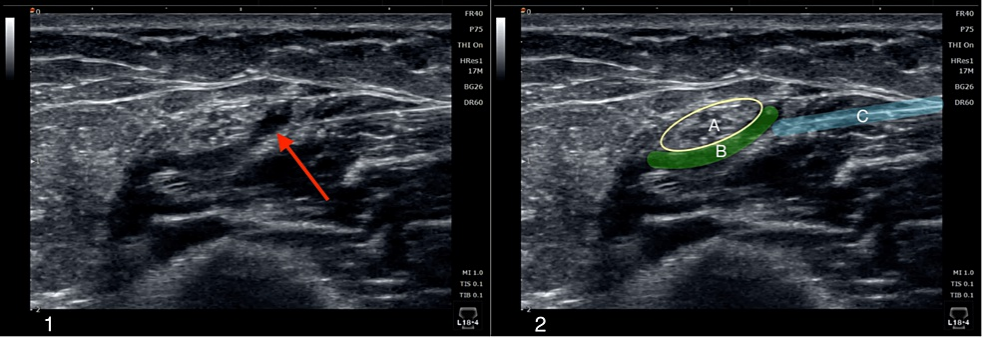
Ultrasound-guided D5W hydrodissection of the right ulnar nerve at the arcade of Struthers. Panel 1: Ultrasound image of the ulnar nerve hydrodissection (red arrow). Panel 2: Identical image of the hydrodissection, with the ulnar nerve encircled in yellow (A); the perineural space highlighted in green (B); and the needle highlighted in blue (C).

The patient deferred formal physical therapy, so a home exercise program was prescribed as well as instructions on postural correction and ergonomic improvements in her workspace. Additionally, nerve hydrodissection of the right elbow was completed a total of three times in a six-week period. A high-frequency linear ultrasound was used to identify the ulnar nerve in short and long axes, and when scanned over the continuity of the ulnar nerve, it demonstrated compression of the ulnar nerve proximal to the cubital tunnel at the arcade of Struthers. Using a 25G 1.5-inch needle, a 10 cc solution of 9 parts dextrose 5% in water (D5W), 0.5 parts of lidocaine 2%, and 0.5 parts Celestone 6 mg/mL was injected 1 cm proximal to the cubital tunnel at the arcade of Struthers into the surrounding area of the ulnar nerve via the short-axis, in-plane approach (Figure 1). The patient maintained a stretching regimen as instructed by the sports medicine physician while taking acetaminophen and ibuprofen as needed, which provided mild symptom relief. Hydrodissection proved successful in progressively alleviating the patient’s symptoms, as the patient reported pain at an 8/10 following the first hydrodissection to a 3/10 after the third hydrodissection. However, the patient remained refractory to complete resolution of symptoms and elected to proceed with platelet releasate treatment.

**Figure 2:**
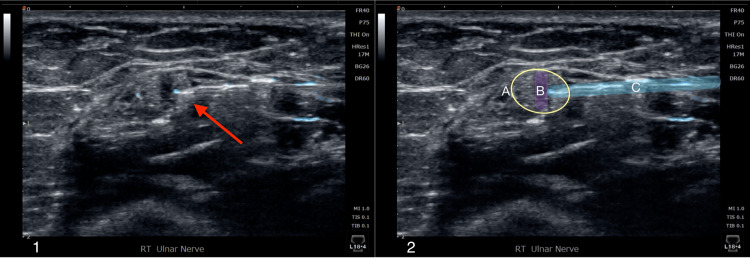
Ultrasound-guided intraneural platelet releasate injection of the right ulnar nerve at the arcade of Struthers. Panel 1: Ultrasound image of the platelet releasate injection within the ulnar nerve (red arrow). Panel 2: Identical image of the injection, with the ulnar nerve encircled in yellow (A); the intraneural space highlighted in purple (B); and the needle highlighted in blue (C).

**Figure 3:**
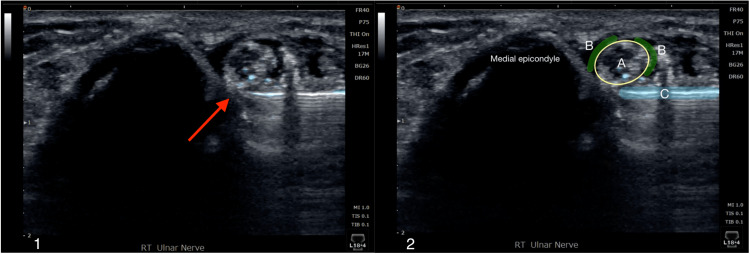
Ultrasound-guided perineural platelet releasate injection of the right ulnar nerve at the cubital tunnel. Panel 1: Ultrasound image of the platelet releasate injection around the ulnar nerve (red arrow). Panel 2: Identical image of the injection, with the ulnar nerve encircled in yellow (A); the perineural space highlighted in green (B); and the needle highlighted in blue (C).

Two weeks following the third hydrodissection, ulnar nerve platelet releasate injection was performed. 120 cc of venous blood was aspirated from the antecubital fossa and spun down using a centrifuge to make PRP. Recombinant thrombin (Recothrom) was then added to the PRP and spun down again in the centrifuge. The platelet releasate was subsequently siphoned off, yielding a total volume of 12 mL of platelet releasate solution. The area of ulnar nerve entrapment at the arcade of Struthers was located using a high-frequency linear ultrasound probe in long and short axis. First, using a 25G 1.5-inch needle, 3 mL of platelet releasate mixed with 0.3 cc of 0.5% ropivacaine was injected intraneurally into the right ulnar nerve at the arcade of Struthers (Figure 2). Then, using a separate 25G 1.5-inch needle, 6 mL of platelet releasate mixed with 0.5 cc of 0.5% ropivacaine was injected perineurally around the ulnar nerve. Lastly, using a separate 25G 1.5-inch needle, 3 mL of platelet releasate mixed with 0.3 cc of 0.5% ropivacaine was injected perineurally around the ulnar nerve at the cubital tunnel (Figure 3). All procedures were completed under ultrasound guidance with image capture. There were no reportable blood losses or complications with the procedures.

Immediately following the platelet releasate injection, the patient reported decreased pain. At the two-week follow-up, the patient reported that symptoms had initially resolved but then returned to pre-procedure levels three days following the procedure. At the six-week follow-up, the patient reported that her elbow pain had improved by 80% compared to before the procedure, and the improvement was still ongoing.

## Discussion

The ulnar nerve begins in the axilla as a continuation of the medial cord of the brachial plexus, originally arising from the C8 and T1 nerve roots of the spinal cord [1]. From the medial cord, the ulnar nerve travels distally through the posterior compartment of the upper arm, passing through the cubital tunnel by the posteromedial aspects of the medial condyle before entering Guyon’s canal once it reaches the hand [1]. Ulnar nerve entrapment can occur at multiple sites, including Guyon’s canal at the wrist or the arcade of Struthers, but most commonly occurs as the nerve travels through the cubital tunnel at the elbow [1]. At the cubital tunnel, ulnar nerve entrapment may be caused by the medial condyle or Osborne’s ligament, which forms the roof of the tunnel [1]. In this case, the patient’s presentation was due to compression from adjacent connective tissue and fascia at the arcade of Struthers, immediately proximal to the ulnar tunnel. A potential site of ulnar nerve entrapment, as the nerve travels from the anterior to posterior muscle compartments of the arm, is the arcade of Struthers, which is a band of connective tissue that is found between the medial intermuscular septum and the medial head of the triceps [1,9]. The patient in this case experienced pain upon palpation at the region where the arcade of Struthers crosses over the ulnar nerve, indicating compression within this structure.

Cadaver studies have illustrated how ultrasound-guided hydrodissection techniques are efficient in separating the ulnar nerve from the medial epicondyle and nearby connective tissue [10]. Hydrodissection has also been demonstrated to reduce pain and improve electrophysiological measurements in small pilot studies of patients with ulnar nerve entrapment [10]. D5W hydrodissection was utilized for this patient to separate the surrounding structures impinging on the ulnar nerve that were causing her symptoms of pain and paresthesia (Figure 1). Given that the patient experienced moderate relief from her symptoms but not complete resolution, nerve damage was most likely present from chronic compression of the ulnar nerve. Further therapy was indicated to treat the damaged nerve as D5W hydrodissection alone did not completely resolve the patient’s symptoms. Consequently, the patient made the decision to undergo nerve platelet releasate therapy.

Platelet releasate is derived as a supernatant from a patient’s own PRP, isolating growth factors essential to tissue regeneration for localized delivery to damaged tissue. PRP, an autologous product of blood-derived platelets in plasma, is a concentration of platelets in plasma that is several times greater than that found in blood at baseline [11]. To obtain PRP, venous blood is first centrifuged in tubes with an anticoagulant to separate its components [7]. Following the collection of the plasma fraction above the red blood cells, thrombin is added as an activating factor to induce platelet degranulation and growth factor exocytosis [7].

While platelet activity is predominantly associated with the initiation of the coagulation cascade, activated platelets possess therapeutic potential in enhancing intrinsic nerve repair processes [12,13]. Bioactive molecules and growth factors released from the alpha-granules of activated platelets such as nerve growth factor, brain-derived neurotrophic factor, and platelet-derived growth factor, have the capability to enhance nerve regeneration [7]. The potential for utilizing these growth factors to repair injured nerves lies in platelet activation and its key drivers. Thrombin, the strongest platelet agonist, activates platelets through protease-activated receptors on their surface via G protein-coupled receptors, facilitating the release of growth factors [13]. By using thrombin to release growth factors from concentrated platelets in PRP, platelet releasate is subsequently formed. This product of activated platelet growth factors thereby holds the potential to expedite nerve recovery. These characteristics of platelet releasate, the supernatant of thrombin-activated PRP, present a compelling case for its use as a treatment modality in relieving pain and restoring function to injured nerves. Like any injection, platelet releasate injection carries a small risk of infection at the injection site or injury to nerves or blood vessels [14]. Adverse effects of PRP injection are rare, and since PRP is prepared from autologous blood, there is minimal risk of an immunogenic reaction [14].

**Figure 4:**
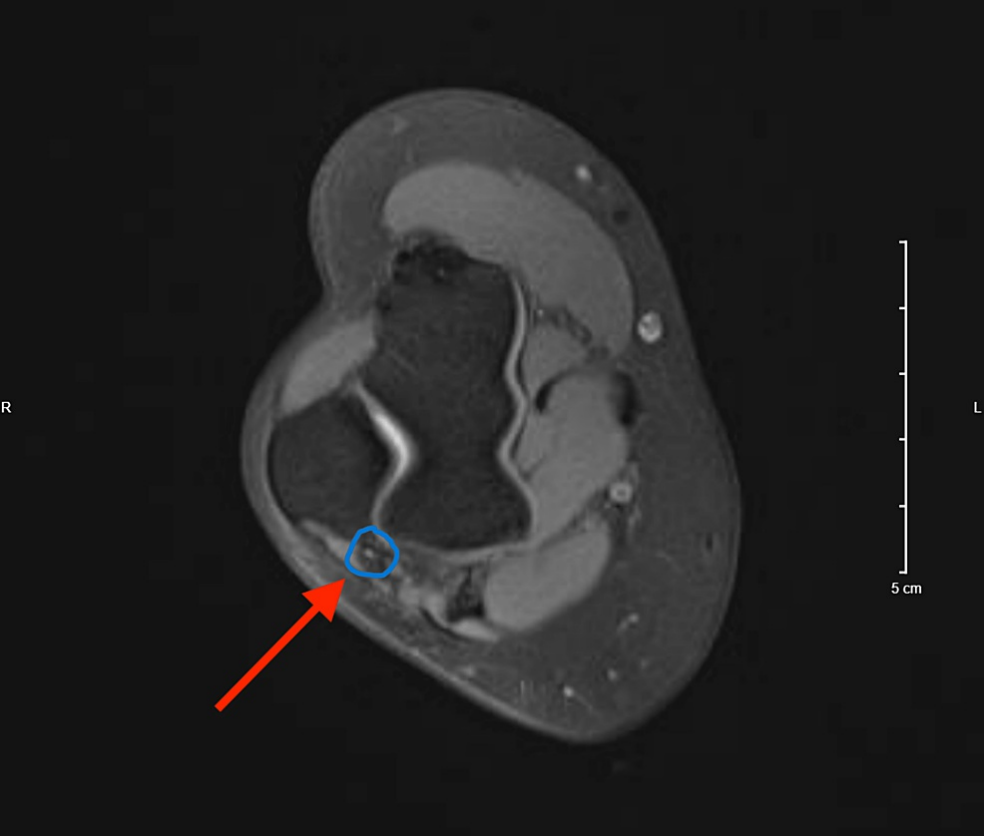
Magnetic resonance imaging of the right elbow six weeks following platelet releasate treatment. Axial view of the right elbow demonstrating a normal-appearing ulnar nerve (red arrow). No structural abnormalities were noted.

Two weeks following the final D5W hydrodissection, platelet releasate was injected intraneurally within and perineurally around the ulnar nerve to accelerate the healing process (Figure 2 and Figure 3). Short-term follow-up with the patient elicited that the nerve platelet releasate therapy completely alleviated symptoms for the first three days following the procedure before the pain progressively returned to pre-procedure levels. This report was consistent with her expected progress of treatment with platelet releasate; follow-up with other patients treated with platelet releasate for nerve compression in the same sports medicine clinic reported similar outcomes, with a resolution of symptoms typically occurring around three-to-six weeks post-procedure. Follow-up at six weeks post-procedure elicited that she had steadily improved but was still not entirely symptom-free. Provocative testing at the elbow as described previously was now negative. Magnetic resonance imaging obtained of the elbow at the six-week mark per the patient’s request demonstrated a normal-appearing ulnar nerve through its course proximal and distal to the elbow, and no other structural abnormality of the elbow was noted (Figure 4).

A probable explanation for the patient’s residual symptoms despite normal imaging and negative provocative testing at the elbow was that there was another point of compression along the course of the ulnar nerve. It was the co-author’s opinion that the patient’s continued symptoms appeared to be from her concomitant neurogenic thoracic outlet syndrome (nTOS). This was further validated by the patient experiencing full relief after a subsequent D5W brachial plexus hydrodissection was performed. Given that nTOS was a contributing factor to the patient’s continued symptoms, it is difficult to ascertain if the platelet releasate therapy was successful in resolving her pain that was due to ulnar nerve entrapment at the elbow. In addition, other factors such as natural healing over time or changes to the patient’s lifestyle following platelet releasate injection could have affected treatment outcomes. However, the negative results from provocative testing, normal imaging findings, and improved elbow pain following platelet releasate therapy provide promising indications that local platelet releasate injection may have effectively promoted the healing of the damaged nerve. These observed findings could potentially be attributed to the platelet releasate therapy creating a favorable environment at the site of ulnar nerve compression at the elbow, accelerating nerve recovery by the mechanisms described earlier.

While current literature has yet to establish nerve platelet releasate injection as a noteworthy treatment modality for ulnar neuritis, this case demonstrates how intraneural and perineural platelet releasate injection may have played a significant role in alleviating pain and restoring function to the patient’s ulnar nerve that was entrapped at the elbow. Additional studies of nerve platelet releasate therapy for related cases may demonstrate its effectiveness in facilitating peripheral nerve recovery.

## Conclusions

This case report investigated the use of ultrasound-guided nerve hydrodissection and platelet releasate injection for treating ulnar neuritis at the elbow due to entrapment. Although D5W hydrodissection proved useful in reducing the patient’s pain and paresthesia, the results of the case suggest platelet releasate injection may have been instrumental in resolving the patient’s localized neuropathy at the elbow. As current literature supports platelet releasate as a key driver of nerve regeneration, it is possible that the platelet releasate injection played a role in reducing the patient’s pain by enhancing the healing response for the injured ulnar nerve. Given the outcome for this patient, this case illustrates the prospect for platelet releasate treatment to continue to be investigated as a potential therapy for ulnar neuritis. Further studies may establish nerve platelet releasate as an efficacious treatment modality for ulnar neuritis and similar peripheral neuropathies.
